# New hallmarks of ageing: a 2022 Copenhagen ageing meeting summary

**DOI:** 10.18632/aging.204248

**Published:** 2022-08-29

**Authors:** Tomas Schmauck-Medina, Adrian Molière, Sofie Lautrup, Jianying Zhang, Stefan Chlopicki, Helena Borland Madsen, Shuqin Cao, Casper Soendenbroe, Els Mansell, Mark Bitsch Vestergaard, Zhiquan Li, Yosef Shiloh, Patricia L. Opresko, Jean-Marc Egly, Thomas Kirkwood, Eric Verdin, Vilhelm A. Bohr, Lynne S. Cox, Tinna Stevnsner, Lene Juel Rasmussen, Evandro F. Fang

**Affiliations:** 1 Department of Clinical Molecular Biology, University of Oslo and Akershus University Hospital, Lørenskog 1478, Norway; 2 Jagiellonian Centre for Experimental Therapeutics (JCET), Jagiellonian University, Krakow 30-348, Poland; 3 Center for Healthy Ageing, Department of Cellular and Molecular Medicine, University of Copenhagen, Copenhagen 2400, Denmark; 4 Institute of Sports Medicine Copenhagen, Department of Orthopedic Surgery, Copenhagen University Hospital – Bispebjerg and Frederiksberg, Copenhagen 2400, Denmark; 5 Molecular Medicine and Gene Therapy, Lund Stem Cell Center, Lund University, Lund, Sweden; 6 Stem Cell Laboratory, UCL Cancer Institute, University College London, London, UK; 7 Department of Clinical Physiology and Nuclear Medicine, Copenhagen University Hospital Rigshospitalet, Glostrup 2600, Denmark; 8 The David and Inez Myers Laboratory of Cancer Genetics, Department of Human Molecular Genetics and Biochemistry, Tel Aviv University School of Medicine P.O.B 39040, Tel Aviv, Israel; 9 Department of Environmental and Occupational Health, University of Pittsburgh School of Public Health, Pittsburgh, PA 15261, USA; 10 Department of Functional Genomics and Cancer, IGBMC, CNRS/INSERM/University of Strasbourg, Equipe labellisée Ligue contre le Cancer, Strasbourg, France; 11 College of Medicine, Center for Genomics and Precision Medicine, National Taiwan University, Taipei City, Taiwan; 12 UK National Innovation Centre for Ageing, The Catalyst, 3 Science Square, Newcastle University, Newcastle upon Tyne, NE4 5TG, UK; 13 Buck Institute for Research on Ageing, Novato, CA 94945, USA; 14 Section on DNA Repair, National Institute on Ageing, Baltimore, MD 21224, USA; 15 Department of Biochemistry, University of Oxford, Oxford OX1 3QU, UK; 16 Department of Molecular Biology and Genetics, Aarhus University, Aarhus 8000, Denmark; 17 The Norwegian Centre on Healthy Ageing (NO-Age), Oslo, Norway; 18 UPMC Hillman Cancer Center, Pittsburgh, PA 15232, USA

**Keywords:** hallmarks of ageing, neurodegeneration, healthspan, longevity, autophagy

## Abstract

Genomic instability, telomere attrition, epigenetic alterations, mitochondrial dysfunction, loss of proteostasis, deregulated nutrient-sensing, cellular senescence, stem cell exhaustion, and altered intercellular communication were the original nine hallmarks of ageing proposed by López-Otín and colleagues in 2013. The proposal of these hallmarks of ageing has been instrumental in guiding and pushing forward research on the biology of ageing. In the nearly past 10 years, our in-depth exploration on ageing research has enabled us to formulate new hallmarks of ageing which are compromised autophagy, microbiome disturbance, altered mechanical properties, splicing dysregulation, and inflammation, among other emerging ones. Amalgamation of the ‘old’ and ‘new’ hallmarks of ageing may provide a more comprehensive explanation of ageing and age-related diseases, shedding light on interventional and therapeutic studies to achieve healthy, happy, and productive lives in the elderly.

## INTRODUCTION

The definition of nine cellular and molecular hallmarks of ageing by López-Otín and colleagues in 2013 [1] provided a contextual framework to guide future ageing research. These hallmarks comprise: genomic instability, telomere attrition, epigenetic alterations, loss of proteostasis, deregulated nutrient-sensing, mitochondrial dysfunction, cellular senescence, stem cell exhaustion, and altered intercellular communication. Recently, these hallmarks have been criticized for being insufficient in serving as a causative paradigm of ageing [2]. Importantly though, they have recently been shown to map to age-related diseases [3]. To address this and to explore potential new hallmarks, a research symposium “New Hallmarks of Ageing” was held in Copenhagen (Denmark) on the 22nd of March 2022, focusing on novel findings and the recontextualization of the nine hallmarks of ageing. This included the discussion of new advances and the future of the field of ageing research.

The symposium contained presentations from the keynote speakers Professors Yosef Shiloh (Tel Aviv University), Vilhelm A. Bohr (National Institute of Ageing), Lynne Cox (University of Oxford), Thomas Kirkwood (Newcastle University), Jean-Marc Egly (Institut de Génétique et de Biologie Moléculaire et Cellulaire), Patricia Opresko (University of Pittsburgh), Erik Verdin (Buck Institute for Research on Ageing) and selected short talks from young and senior researchers, and ended with a panel discussion with some of the key speakers of the event.

The panel stressed the importance of progress in the field, as ageing is the primary risk factor of many major human diseases. It was highlighted that increasing average lifespan over the last decades is one of the most remarkable human accomplishments, but that this success has led to a different, challenging problem, namely the ever-increasing number of chronically ill patients suffering from age-related diseases, and the resulting toll on individuals and society. Understanding the mechanisms of the ageing process will therefore be pivotal to treat the root cause of multiple age-related diseases.

**Figure 1:**
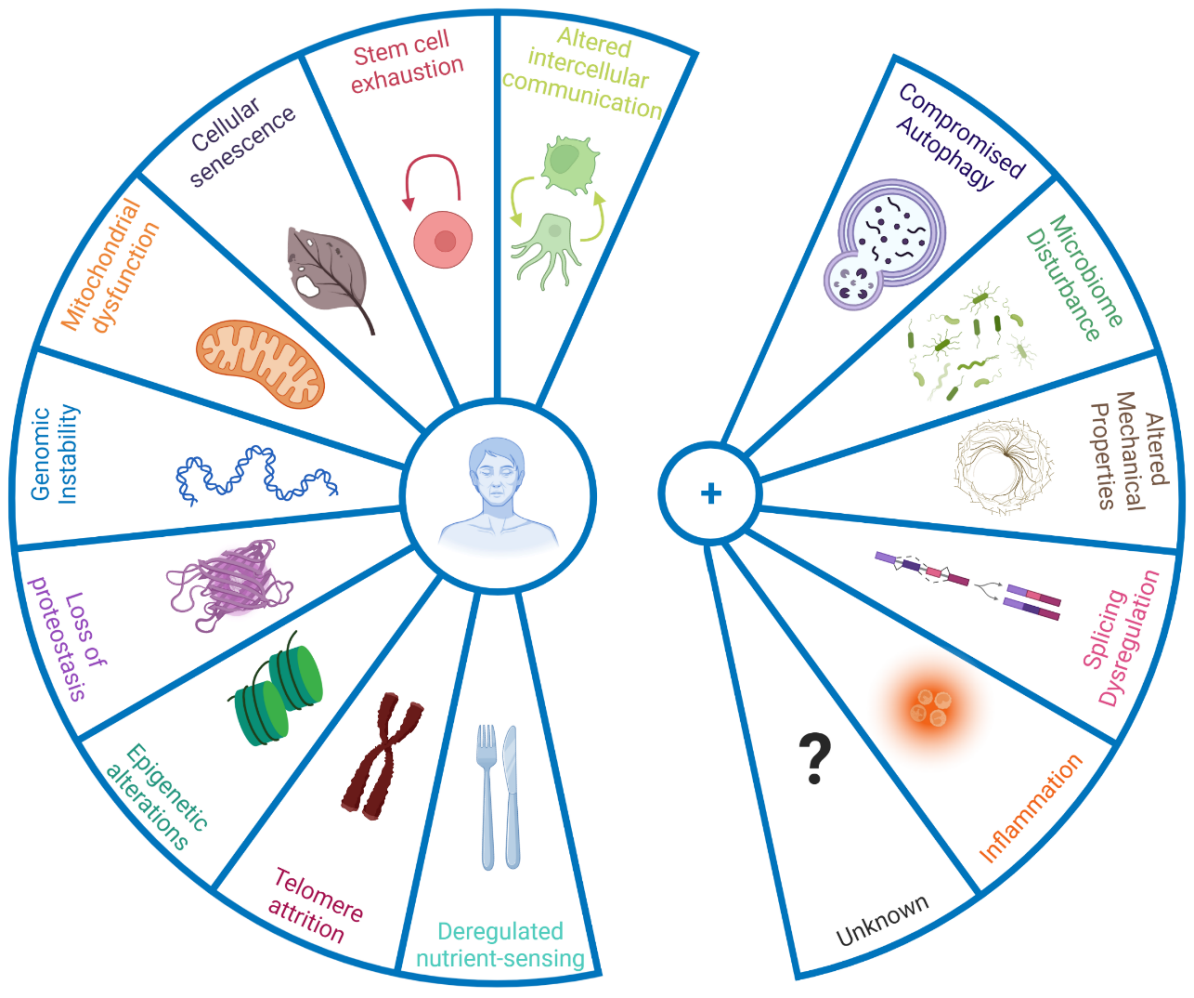
**New hallmarks of ageing.** The figure enumerates the original hallmarks of ageing plus the five new proposed hallmarks that were discussed in the symposium. To qualify as a hallmark, the processes should change with biological age not simply in a correlative manner, but have a causal role. Hence interventions that address the hallmarks should, at the very least, halt further detrimental aspects of ageing, and preferably improve phenotypes associated with ageing.

The panellists emphasised that only taking a limited number of defined hallmarks into account might also halt progress on processes relevant to ageing but not currently defined as hallmarks. The panellists thereby discussed the inclusion of new hallmarks to the current list (Figure 1), based on new evidence underpinning their role in the ageing process. Some discussed additions were compromised autophagy, dysregulation in RNA splicing, inflammation, loss of cytoskeleton integrity, and disturbance of the microbiome (Box 1).

**Box 1:** New hallmarks of ageing.

**Compromised autophagy** is observed in numerous ageing conditions including neurodegeneration and immunosenescence [8, 9]. Importantly activation of autophagy can increase mouse lifespan [10], and even improve immune response to vaccination in older humans by overcoming immunosenescence [11]. While originally considered under hallmark ‘altered proteostasis’, autophagy regulates a number of other hallmarks of ageing such as DNA repair and nutrient sensing/metabolism [12], and hence it was proposed to be categorised as an integrative hallmark.
**Dysregulation of RNA processing** has been noted in human ageing population studies [13] while interventions that appear to reverse senescent phenotypes act at least in part by restoring youthful patterns of splicing factor expression [14]. Similarly, alternative polyadenylation of mRNAs, already known to contribute to cancer [15] is altered with ageing and may contribute to senescence [16]. Such changes in RNA processing add an additional layer of gene expression control over those of genome integrity, transcriptional efficacy and epigenetic regulation that are already known to change during biological ageing.
**Microbiome disturbances:** recent advances in next generation sequencing technologies have allowed the identification of notable changes in the gut microbiome with age [17], pointing in particular to shifts in microbial populations and loss of species diversity. Together with age-associated loss of structural integrity of the gut and other barriers (e.g. blood brain barrier), this shift in microbial populations can drive inflammation.
**Altered mechanical properties** applies both to cells and to the extracellular milieu. For example, fibroblast senescence is accompanied by a major change from a mobilizable pool of actin that can be readily polymerised and depolymerised during cell motility, to stable stress fibres of f-actin anchored through focal adhesions to the substrate [18], which is particularly marked in cells from patients with premature ageing syndromes [19] and which is likely to impact on cell motility and cell-cell communication. Motility changes are of major relevance in innate immune system ageing, where neutrophils from old donors cause significant tissue damage on migration to sites of inflammatory signalling; modification of the small G protein signalling pathways that regulate such cytoskeletal motility through treatment with statins greatly improves older neutrophil action *in vitro* and results in highly significant increases in 6 month mortality follow up from older adults admitted to the ICU with pneumonia [20]. The nucleoskeleton is also altered during ageing, with the nuclear lamina becoming destabilised, with concomitant extrusion of chromatin into the cytoplasm as CCFs which trigger the SASP in senescence [21]. Importantly, the nuclear lamina is highly defective in Hutchison-Gilford progeria [22] and clinical trials of farnesyl transferase inhibitors that restore NE integrity increase patient lifespan [23]. Finally, extracellular matrix also changes with ageing, which greatly alters cell behaviour [24]. Increased rigidity and loss of elasticity, for example arising through glycation cross-links between collagen molecules, can lead to multiple age-related disease states such as hypertension with concomitant kidney and neurological defects – such cross-linking may contribute to the accelerated ageing seen in patients with diabetes [25]. The field of mechanobiology and its intersection with ageing is thus very promising in terms of ‘rejuvenation’.
**Inflammation:** Inflammageing, age-dependent chronic inflammation, is implicated in a wide range of age-related diseases [26]. Ageing correlates with high, levels of inflammatory mediators in the blood, such as IL-1, IL-6, C-reactive protein, IFNα, and several others [27]. Originally inflammation was considered part of the hallmark 'altered intercellular communication', however it could be considered on its own merit, due to its large contribution to the ageing process and its cross-play with other hallmarks such as cellular senescence and the newly proposed gut microbiota [26, 28].

These seem particularly timely, but there will likely be a continuing, ongoing discussion of the ageing hallmarks. In the view of the panel, the framing in terms of hallmarks offers a useful simplification of a very diverse field, especially for newcomers, but it should not be accepted as a sufficient platform for research on the root causes of the ageing process. Nonetheless, the hallmarks have proven useful in identifying targets for intervention in age-related diseases, e.g. therapies targeting the accumulation of senescent cells, with promising results in preclinical models [4–6] and early stage human clinical trials [7].

Furthermore, the panel discussed that the hallmarks of ageing have greater value when viewed as a network rather than individual processes, and more focus on the interconnectedness of different hallmarks is urgently needed, together with wider adoption of systems biology approaches. Recognition that the ageing process results in progressive loss of homeostasis will also be informative for research aiming to restore a youth-like balance, by for example restoring youthful gene/protein expression levels and patterns and/or judicious supplementation of metabolites that have become depleted with age.

When discussing the future of ageing research, it was highlighted that the field as a whole has not yet fully delivered. The ageing process is multifaceted and multifactorial, and the panellists stressed that the classical approach of focussing on single pathways or even individual hallmarks might fail to capture the complexity an interconnectedness of ageing. Furthermore, translational work to in humans should have a stronger focus, especially to evaluate the safety profile of possible interventions (dose, concentration, intervals, etc.).

The work presented in this symposium consisted of some of the latest findings in the field in the context of reevaluated hallmarks of ageing. Here we group and summarize the presentations by overall theme.

## Theories of ageing and cellular senescence

**Figure 2:**
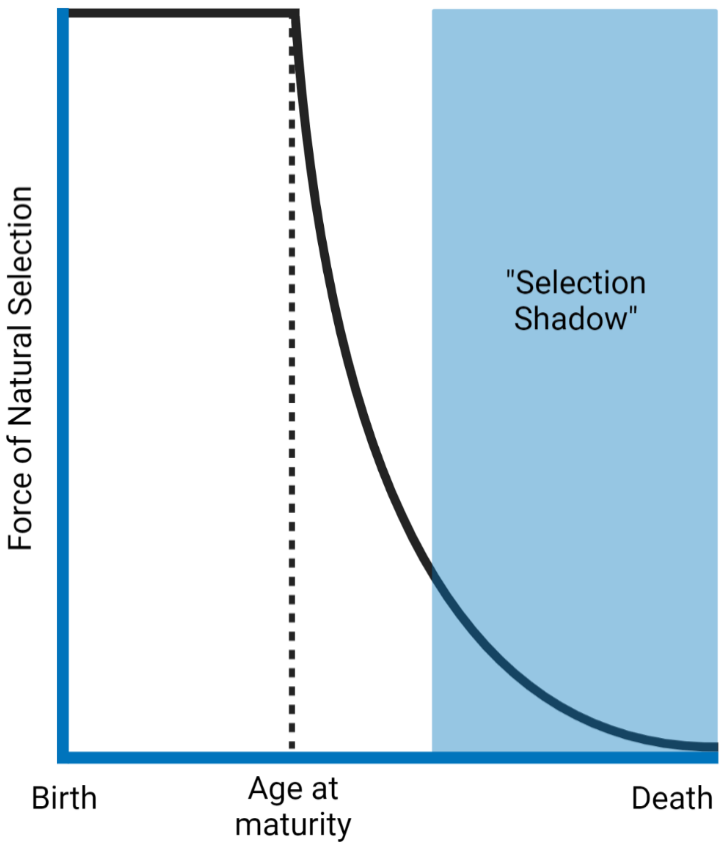
**The force of natural selection in ageing.** The force that selects different traits declines as a function of age after reproductive age. Natural selection benefits survival to maturity and reproduction. Therefore, traits that present harmful effects late in life (selection shadow) will likely have already been passed to the next generation. The figure was generated based on the reference [34].

Professor Thomas Kirkwood (University of Newcastle) began his talk examining evolutionary theories of ageing. The theory of the disposable soma [29] describes that an organism has limited resources, which it has to allocate strategically. For instance, allocating resources towards growth may lead to less investment in DNA repair. Secondly, he highlighted the antagonistic pleiotropy hypothesis [30], which outlines that genes leading to harmful effects in old age might not be selected against due to an advantage in early life. Both of these theories derive from the principle that the force of natural selection declines with increasing age (Figure 2).

From here Professor Kirkwood focused on the hallmark of cellular senescence. Together with Dr. Axel Kowald, they analyzed existing data on the use of senolytics in mice using the Gompertz model [31]. While the median lifespan increased by a striking 27%, the maximum lifespan saw marginal increase (2.8%). However, the rate of ageing seems to accelerate with senolytic treatment. This is compatible with the hypothesis that senescent cells can have beneficial effects, such as wound healing, proposed several years ago [32]. This gives further reason for the evolution of senescence-associated secretory phenotype (SASP).

Through modelling studies, Kirkwood and Kowald proposed that while senolytics may cause a short-term improvement in survivorship, they may impair the capacity of the organism to repair itself, which might lead to a faster accumulation of senescent cells when the treatment concludes. This highlights the importance of further analysis to fully understand the potential benefits and side effects of senolytic therapy.

While the importance of cellular senescence in ageing is well established [33], how this process is interconnected to other hallmarks of ageing remains elusive. In her presentation, Professor Lynne Cox (University of Oxford), explored these relationships, emphasising the importance of cellular senescence and deregulated nutrient sensing. She and others found that the inhibition of mTOR, a central regulator of metabolism and growth, leads to an improvement in senescent phenotypes and a delay in cellular senescence [18]. Similarly, mTOR inhibition alleviated the hallmark of mitochondrial dysfunction, with improved mitophagy and reduced mitochondrial load [18]. Senescence-associated changes in the cytoskeleton - proposed as a component of new hallmarks of ageing ‘altered mechanical properties’ (Figure 1) – are intrinsically linked with altered cell-cell communication, with an increase in cell contacts and a higher transfer of mitochondria through tunnelling nanotubes occurring between cells during cellular ageing. Both phenotypes were ameliorated after mTOR inhibition [35]. Finally, mTOR inhibition also alleviated markers of genomic instability and telomere attrition in senescent cells, demonstrating the value of mTOR inhibitors as geroprotectors, consistent with numerous findings from preclinical model organisms [36]; new studies in dogs (DogAgeing Project) and Humans (PEARL Trial) will therefore be particularly informative.

## New insights into telomeres and cellular senescence

Telomere attrition is a central feature of cellular senescence. Still, the effects of stress on telomeres and subsequent premature senescence remain unclear. One proposed model is that oxidative stress induces premature senescence through the shortening of telomeres. To investigate this question, Professor Patricia Opresko (University of Pittsburgh) presented a new cellular tool to precisely induce oxidative stress in the form of 8-oxoguanine in telomeres [37]. Indeed, chronic oxidative stress in immortalized cell lines leads to shortening of telomeres, impaired growth, and genomic instability, while acute stress showed little effect [37].

However, this effect was drastically different in non-disease cells. New data from Opresko’s lab shows that a single dose of oxidative stress at telomeres was enough to inhibit cell growth and induce premature senescence. However, this p53-dependent form of premature senescence was importantly not accompanied by a shortening of telomeres, but rather by telomere fragility. The telomeres were furthermore hypersensitive to replication stress caused by 8-oxoguanine, and it was hypothesized that stalling of replication could explain the arrest in cell growth and premature senescence [38].

## Inflammation, NAD+ and ageing

Cellular senescence, associated with the pro-inflammatory SASP, interlinks with inflammation, which is emerging as an important driver of organismal ageing [39]. Dr. Helena Borland (University of Copenhagen) showed in her short-talk how the compound Urolithin A modulates the cGAS-STING pathway by increasing STING expression, thus amplifying innate immune activation by stimulation. Urolithin A has been reported to possess anti-inflammatory properties for example by reducing activation of another regulator of innate and adaptive immunity, NF-κB [40]. The presented data thus shows that the effects of Urolithin A is context dependent, which is relevant in modulation of the immune response and inflammation.

Professor Eric Verdin (Buck Institute for Research on Ageing) presented new findings regarding CD38, one of several NAD^+^ consuming enzymes, that is highly expressed on immune cells. NAD^+^ has become the focus of intense interventional studies over the last decades, since it decreases in several tissues with age and in age-related diseases [41], while supplementation with NAD^+^ precursors has shown potential to prevent or slow the progression of various age-related diseases, for which there are several on-going clinical trials [42].

Professor Verdin showed that, while NAD^+^ levels fall, the expression of CD38 in tissue-resident macrophages increases during ageing. NAD^+^ consuming enzymes stand in competition with each other, and the subsequently higher demand for NAD^+^ due to overexpressed CD38 might inhibit other NAD^+^ consuming enzymes critical for geroprotection. Indeed, knock-out of CD38 fully rescued the age-dependent decrease in NAD^+^ levels in adipose tissue [43]. Taking a closer look at the expression pattern of CD38 the Verdin lab found that there is an age-related increase in the expression of CD38, specifically in resident pro-inflammatory M1 macrophages, supporting the role of inflammation during ageing (Figure 1). The expression of CD38 in macrophages furthermore increased in response to pathogen-associated molecular patterns, but not to damage-associated molecular patterns.

Connecting back to senescence, Professor Verdin's group found that the inflammatory SASP increased CD38 expression in mouse embryonic fibroblasts. In addition, SASP cytokines IL-6, TNF-α, and IL-10 were also capable of inducing CD38 expression. Taken together, CD38 emerges as a novel target to ameliorate age-related diseases, by counteracting inflammation and bringing the dysregulated NAD^+^ system back to balance.

Among the protective enzymes that compete with CD38 for available NAD^+^ are the family of poly ADP-ribose polymerases (PARPs). Professor Vilhelm A. Bohr (National Institute on Ageing) presented new data on the DNA damage response enzyme PARP1 related pathways. While the role of PARP1 in nuclear DNA repair and maintenance is well established, a possible role in mitochondria is controversial. Professor Bohr suggested that PARP1 might be present and functional in mitochondria, at least in small amounts.

## Mitochondrial dysfunction

Mitochondrial dysfunction is a central node in the network of hallmarks of ageing and is known to be a strong driver of stem cell ageing [44–46]. Age-related changes in hematopoietic stem cells (HSCs), that are responsible for life-long production of all blood lineages as well as stem cell pool maintenance, cause predisposition to myeloid malignancies, adaptive immune compromise and anaemia. Functional restoration of ageing tissue-resident stem cells is therefore of high interest. Dr. Els Mansell (Lund University and University College London) showed that mitochondrial membrane potential (ΔΨm) decreases in aged hematopoietic stem cells (HSC) but that a small fraction of ΔΨm ^high^ HSCs remain present in the bone marrow of old mice. Strikingly, her data reveal that ΔΨm is a stronger determinant of the transcriptional state of HSCs than chronological age. In addition, enhancement of ΔΨm through mitoquinol treatment resulted in rescue of metabolic, transcriptional and functional parameters of old HSC. Importantly, mitochondrial treatment of old mice corrected the age-related myeloid bias in the peripheral blood and improved the engraftment potential of old HSCs [47].

A major cellular process to maintain the quality of mitochondria is mitophagy; ageing and genetic factors that retard mitophagy could lead to accumulation of damaged mitochondria in cells and tissues, a major cause/risk factor of different diseases, including neurodegenerative diseases [48–50]. The importance of dysfunctional mitochondria in age-related diseases was underpinned by Dr. Shuqin Cao (Akershus University Hospital and University of Oslo), who presented data on a novel inducer of mitophagy extracted from *Passiflora edulis* (passion fruit) ameliorating phenotypes of Alzheimer’s disease in *C. elegans* and cell models [51]. Furthermore, Dr. Jianying Zhang (University of Oslo and Central South University) showed that the levels of a key autophagy regulator in human cerebrospinal fluid can be used as a potential biomarker to predict the clinical severity and cognitive trajectories in Alzheimer’s disease.

## Premature ageing and DNA repair

Professor Bohr presented the short-term use of NAD^+^ supplementation in another age-related pathology, namely presbycusis, or age-related hearing loss. His group has previously shown that supplementation with the NAD^+^ precursor nicotinamide riboside (NR) partially prevents hearing loss and improves cochlear health in mouse models of Cockayne syndrome (CS), an accelerated ageing disease [52]. However, whether this was translatable to age-related hearing loss in non-disease models was previously unknown. Here, Professor Bohr’s group showed that the treatment of mice with NR was capable of restoring NAD^+^ in the cochlea of aged mice to the levels found in young mice. Even more strikingly, NR treatment limited the progression of hearing loss in ageing mice while stopping the progression of hearing loss in old mice. Based on distortion product otoacoustic emissions and wave-form analysis, the group hypothesize that the benefit may arise due to an improvement in the inner hair cells, auditory nerves and/or synaptic transmission.

Another characteristic of CS is dysfunctionality in a sub-pathway of nucleotide excision repair (NER), specifically transcription-coupled repair. Professor Jean-Marc Egly (Institut de Génétique et de Biologie Moléculaire et Cellulaire) discussed that CS complementation group A and B (CSA/CSB), the disease-defining proteins in CS, may target Pol II when at a DNA lesion to recruit the NER machinery for repair. However, upon UV stress, cells from CS patients stall in transcription without subsequent recovery. Professor Egly’s group found that this stall of transcription was sustained by the incapability of mutant CSA and CSB to promote degradation of the chromatin-bound protein ATF3 [53], which suppresses thousands of genes as a UV stress response. On the contrary, in wild-type cells, ATF3 has cleared from chromatin 24h post UV treatment. In addition, Professor Egly also presented data on the role of CSA and CSB in the regulation of cell division [54]. CSA and CSB seem to be recruited to the midbody of the cell, recruit ubiquitin ligases CUL4 and MDM2, and promote the degradation of PRC1 to assist cell division. These findings do not only show a potential target for therapy, but may also be useful as a potentially simple clinical marker for CS diagnosis.

An additional premature ageing disease is the genome instability disorder, ataxia-telangiectasia (A-T). A-T is caused by null mutations in the *ATM* gene, which encodes the homeostatic, multi-functional protein kinase, ATM. A major function of ATM is orchestrating the cellular response to double-strand breaks in the DNA, but it is also involved in regulating the cellular redox balance, mitochondrial homeostasis and several other metabolic circuits. The premature ageing component of A-T is studied by Professor Yosef Shiloh (Tel Aviv University). His work is currently focused on the premature senescence that is a phenotypic hallmark of cultured primary fibroblasts from A-T patients. In view of ATM’s role in the cellular response to reactive oxygen species, this phenotype was examined in cells grown under physiological oxygen level (3%) instead of ambient level (21%). However, the difference in senescence pace between A-T and non-disease cells was maintained. Furthermore, transcriptomic analysis showed strikingly similar dynamics of gene expression patterns in A-T fibroblasts senescing at early passage levels and non-disease cells senescing much later. This observation suggests that the same replicative senescence occurs in both genotypes, but this process is inherently accelerated in A-T cells, highlighting the role of genome stability in determining senescence pace, with implications on organismal ageing.

A-T is an autosomal recessive disease but the phenotypic effects of heterozygosity for A-T mutations has been a focus of interest and some controversies for many years, particularly with regard to cancer predisposition conferred by this genotype. To this end, Professor Yosef Shiloh presented data from an ongoing epidemiological study, in which a large Israeli cohort of A-T carriers and controls has been followed since the late 1990s. As these individuals are advancing in age, a moderate but significant increase of cancer occurrence is being observed among the carriers compared to controls suggesting mild predisposition to many types of cancers among the carriers. Initial findings also suggest a moderate increase in cardiovascular diseases to be associated with this genotype. Since A-T carrier frequency may reach 1% – 3% in various populations these findings highlight the role of genome stability in common human morbidity. Collectively, sequence alterations in many genes associated with maintenance of genome integrity may implicate significantly of ageing and ageing-associated morbidity in the general population.

## Cardiovascular, cerebrovascular and muscular pathologies of ageing

Most of the preclinical experimental studies on the mechanism of endothelial dysfunction are experimentally separated from vascular ageing, even though the latter represents the major driver of deterioration of endothelial function, vascular stiffness and of cardiovascular diseases in humans. In this context, Professor Stefan Chlopicki (Jagiellonian University) highlighted the importance of the accelerated age-dependent development of endothelial dysfunction in the pathogenesis of cardiovascular disease based on experimental studies performed with the use of the unique MRI-based *in vivo* methodology to characterize endothelial function in murine models of dyslipidaemia and vascular ageing [55, 56].

The cerebrovascular function also declines with age. Especially the cerebrovascular reactivity to vasodilating stimuli and the perfusion modulation is affected [57], with severe decline being observed in diverse neurodegenerative diseases. Dr. Mark Vestergaard (Rigshospitalet) showed that a correlation between subtle cognitive deficits and an inhibited cerebrovascular response to neuronal activation can be observed before the rise of neurodegeneration [58].

Currently, lifestyle factors like diet and exercise are still the best protectors against age-related diseases. On this note, Dr. Casper Soendenbroe provided novel evidence that even physical activity performed at a recreational level offers some protection against the debilitating effects of ageing [59]. Specifically, lifelong exercising men had preserved levels of muscle stem cells and improved neuromuscular innervation, which culminated in a clinically relevant preservation of muscle function.

## Concluding remarks

The symposium hosted several established researchers and young scientists in the field to present and discuss the latest findings in age-related research, and how this work connects to established and newly proposed hallmarks of ageing. The symposium was held as a hybrid event, with both physical and virtual attendance. The large international audience that was amassed highlights the growing interest in the field.

The presented data showcased novel research at the forefront of the field, with a focus on a possible increase in healthspan and the amelioration of age-related diseases. Here, both the possible clearance or delay of senescent cells as well as possible interventions in the NAD^+^ system were discussed. While both of these approaches are promising, they are not without limitations.

At this point, tremendous progress has occurred, but a unified theory of ageing that can fully explain the process is still missing, and many open questions remain, both on a cellular and organismal level. Whether it is possible to target the ageing process at its core, or whether a combination of approaches is needed to target the aspects encompassing ageing, remains to be solved in the future.
